# Healthcare-associated infections: bacteriological characterization of the hospital surfaces in the University Hospital of Abomey-Calavi/so-ava in South Benin (West Africa)

**DOI:** 10.1186/s12879-018-3648-x

**Published:** 2019-01-07

**Authors:** F. Cyr Doscoph Afle, Alidéhou Jerrold Agbankpe, Roch Christian Johnson, Olivia Houngbégnon, Sègbè Christophe Houssou, Honoré Sourou Bankole

**Affiliations:** 1 Interfaculty Center of Training and Research in Environment for Sustainable Development, University of Abomey-Calavi, 01 PO Box 1463, Cotonou, Benin; 2 Research Unit in Applied Microbiology and Pharmacology of Natural Substances, Research Laboratory in Applied Biology, Polytechnic School of Abomey-Calavi University, University of Abomey-Calavi, 01 PO Box 2009, Cotonou, Benin; 3 Bacteriology Laboratory of the Ministry of Public Health, 01 PO Box 418, Cotonou, Benin; 4 Faculty of Human Sciences, University of Abomey Calavi, Cotonou, Benin

**Keywords:** Healthcare-associated infections, Bacteriological characterization, Hospital environment, Benin

## Abstract

**Background:**

Healthcare-associated infections have become a public health problem, creating a new burden on medical care in hospitals. The emergence of multidrug-resistant bacteria poses a difficult task for physicians, who have limited therapeutic options. The dissemination of pathogens depends on “reservoirs”, the different transmission pathways of the infectious agents and the factors favouring them. Contaminated environmental surfaces are an important potential reservoir for the transmission of many healthcare-associated pathogens. Pathogens can survive or persist in the environment for months and be a source of infection transmission when appropriate hygiene and disinfection procedures are inefficient. The aim of this study was to identify bacterial species from hospital surfaces in order to effectively prevent healthcare-associated infections.

**Methods:**

Samples were taken from surfaces at the University Hospital of Abomey-Calavi/So-Ava in South Benin (West Africa). To achieve the objective of this study, 160 swab samples of hospital surfaces were taken as recommended by the International Organization for Standardization (ISO 14698-1). These samples were analysed in the bacteriology section of the National Laboratory for Biomedical Analysis. All statistical analyses were performed using SPSS Statistics 21 software. A Chi Square Test was used to test the association between the Results of culture samples and different care units.

**Results:**

Of the 160 surface samples, 65% were positive for bacteria. The frequency of isolation was predominant in Paediatrics (87.5%). The positive samples were 64.2% Gram-positive bacteria and 35.8% of Gram-negative bacteria. *Staphylococcus aureus* predominated (27.3%), followed by *Bacillus* spp*.* (23.3%).

The proportion of other microorganisms was negligible. *S. aureus* and *Staphylococcus spp.* were present in all care units. There was a statistically significant association between the Results of culture samples and different care units (*χ*2 = 12.732; *p* = 0.048).

**Conclusion:**

The bacteria found on the surfaces of the University Hospital of Abomey-Calavi/So-Ava’s care environment suggest a risk of healthcare-associated infections. Adequate hospital hygiene measures are required. Patient safety in this environment must become a training priority for all caregivers.

## Background

Healthcare-associated infections (HAI) are a public health problem due to their high morbidity and mortality rates and subsequent economic consequences [1]. Pathogens responsible for these infections are varied and may have an endogenous or exogenous origin [2]. Despite advances in healthcare safety, the hospital environment can contribute to the spread of pathogens by transferring bacteria between patients and the environment [3]. Healthcare workers should be aware of the role of environmental contamination in the Intensive Care Unit and consider it in the broader perspective of infection control measures and stewardship initiatives [4]. Thus, pathogens can contaminate the surfaces of the hospital environment at concentrations sufficient for transmission from the hands of the nursing staff, or survive persist despite the disinfection of the environment [5]. Prior contamination of the patient environment is a factor in the acquisition of Healthcare-associated infections [6]. The microbial ecology of the care units remains a known risk factor for these infections [3].

In Benin, efforts are being made to fight healthcare-associated infections, but a study of patients admitted to a Benin hospital in 2012 reported a patient infection frequency of 9.84% [7]. In the fight against this health problem, it is important to carry out microbiological controls to identify the sources of pathogens in the hospital environment and with the infectious risk [8]. Therefore, knowledge of the microbiological contamination of the surfaces surrounding a patient provides information on the activity in the room, the presence of nosocomial pathogens and the quality of the disinfection [8, 9]. It is in this context that we aimed to determine the bacterial ecology on surfaces or even medical devices at the University Hospital of Abomey-Calavi/So-Ava in South Benin (West Africa).

## Methods

In order to identify the bacterial species contaminating the hospital environment, an analytical cross-sectional study was carried out on surface samples.

### Study framework

This work spanned two months, from February to March 2017. It was carried out in the University Hospital of Abomey-Calavi / So-Ava in South Benin (West Africa). CHUZAS has about 100 beds, and is located in a southern Benin city of a population of 656,358 inhabitants [10]. The samples were taken from various sites in the hospital environment, in various departments (surgery, maternity, paediatrics, medicine, emergency, operating theatre, recovery room). Bacteriological testing was carried out at the National Laboratory of Biomedical Analysis of the Ministry of Health (Benin).

### Equipment

Conventional materials used in bacteriological diagnosis were used. Eosin Methylene-Blue (EMB), Chapman, Mueller Hinton Agar (MH) and API 20 E gallery were used.

### Sample collection

The 160 samples were taken at different environmental sites according to the care units (Table 1). The areas sampled were: surgery tables; medical instrument tables; neonatal resuscitation tables, reusable electrosurgical patient plate, blood pressure cuff, oxygen mask, access door latches, care trolleys, patient examination tables, stethoscope pavilion, hospital bed sheets and the ground.

**Table 1:** Results of culture samples

Services	Positive results	Number
Operating theatre	18 (47.4%)	38
Recovery room	13 (12.2%)	18
Maternity	23 (71.9%)	32
Medicine	12 (75%)	16
Paediatrics	14 (87.5%)	16
Surgery	14 (70%)	20
Emergency services	10 (50%)	20
Total	104 (65%)	160

104 samples were positive for bacteria

Greatest frequency of bacterial isolation observed in paediatrics (87.5%)

Lowest proportion of bacterial isolation found in the operating theatre (47.4%)

The samples were taken in the morning one hour after cleaning and disinfection of the room. For some care units (surgery room), the samples were taken after disinfection of the room without prior use. Swabs were taken according to ISO 14698-1 [11].

The sterile swabs were moistened with sterile distilled water and passed in parallel striations on the surface by turning them slightly, then in perpendicular striations on the same zones.

Subsequently, the swabs were returned to their protective cases and transmitted to the laboratory within one hour. We obtained a total of 160 samples from the 64 selected sites across the 7 investigative care units. The number of samples varied between 2 and 4 per site.

### Isolation

The samples were emulsified in haemolysis tubes with 5 ml of Brain Heart broth (BHB) and incubated at 37 °C for 24 h. Bacterial growth manifested in the form of turbidity. Each sample showing turbidity was streaked on Eosin Methylene-Blue, Chapman and MH medium and incubated at 37 °C for 24 h.

### Identification

Gram staining was carried out directly on one isolated colonies. One characteristic Colonies with Gram-negative bacilli (GNB), Gram-positive bacilli (GPB) and Gram-positive cocci (GPC) were then selected.

After purification, biochemical identification of GNB was carried out by seeding the API 20 E gallery. For the identification of *S. aureus*, catalase, coagulase and DNase tests were carried out on Gram-positive cocci. Catalase, oxidase, and urease tests were also performed on sporulate large Gram-positive bacilli for the identification of *Bacillus* spp.

### Statistical analysis

All statistical analyses were performed using SPSS Statistics 21 software. A Chi Square Test was used to test the association between the Results of culture samples and different care units.

## Results

### Result of samples

Of the total 160 samples analysed, 65% (*n* = 104) were positive for bacteria (Table 1).

The distribution by services is presented in Table 1. The greatest frequency of bacterial isolation was observed in paediatrics (87.5%) and the lowest proportion was found in the operating theatre (47.4%). There was a statistically significant association between the Results of culture samples and different care units (*χ*2 = 12.732; *p* = 0.048).

### Biochemical identification

The study of biochemical characteristics revealed that of the total 150 species identified, 63.3% (*n* = 95) of the isolates were Gram-positive bacteria, and 55 36.7% (*n* = 55) were Gram-negative bacteria.

### Distribution of gram-positive bacteria

**Fig. 1:**
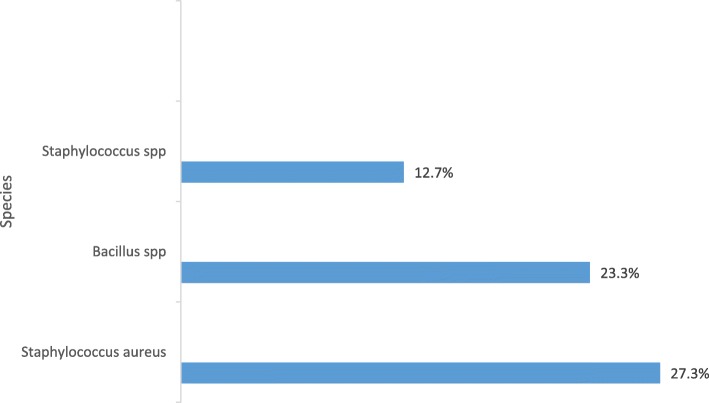
Species of Gram-positive bacteria. *Staphylococcus aureus* have the greatest frequency (27.3%). *Staphylococcus* spp. have the lowest frequency (12.7%)

Gram-positive bacteria consisted of 3 different species (Fig. 1). The most frequent were *Staphylococcus aureus* 27.3% (*n* = 41).

### Distribution of bacterial species belonging to Enterobacteriaceae family

There were 4 genera that constituted 39 strains of bacteria from Enterobacteriaceae groupe identified in the study (Table 2).

**Table 2:** Distribution of bacterial species belonging to Enterobacteriaceae family

Genus	Species	Number of species	% of species	Number of genus	% of genus	Total number of Enterobacteriaceae
Klebsiella	*Klebsiella pneumoniae*	02	1.3	05	3.3	39
	*Klebsiella oxytoca*	02	1.3			
	*Klebsiella ornithinolytica*	01	0.7			
Serratia	*Serratia ficaria*	08	5.3	10	6.7	
	*Serratia marcescens*	01	0.7			
	*Serratia rubidaea*	01	0.7			
Pantoea	*Pantoea spp. 2*	03	2.0	09	6.0	
	*Pantoea spp. 3*	02	1.3			
	*Pantoea spp. 4*	04	2.7			
Other germs	*Escherichia coli*	11	7.3	15	10.0	
	*Citrobacter freundii*	02	1.3			
	*Shigella spp.*	01	0.7			
	*Kluyvera spp.*	01	0.7			

The most frequent were *Escherichia coli* 7.3% (*n* = 11), followed by *Serratia ficaria* 5.3% (*n* = 08).

### Distribution of non-fermenting gram-negative bacilli NFGNB

**Fig. 2:**
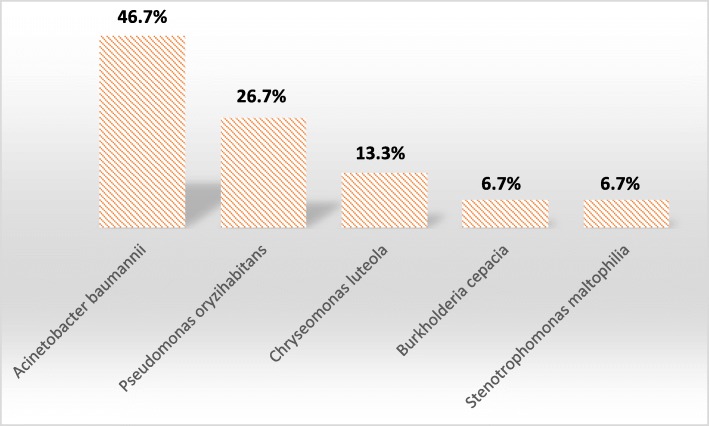
Distribution of NFGNB. *Acinetobacter baumannii* have the greatest frequency (46.7%)

The Non-Fermenting Gram-Negative Bacilli (NFGNB) strains consisted of 15 species. *Acinetobacter baumannii* and *Pseudomonas oryzihabitans* constituted 46.7% (*n* = 7) and 26.7% (n = 4), respectively (Fig. 2).

### Repartition of species identified according to care units

Species identified in survey services are presented in Table 3. *Staphylococcus aureus* (23.3%) *and Staphylococcus spp* (12.7%) were present in all care units (Table 3).

**Table 3:** Distribution of species identified according to care units

Species	Number of species according to care units							Number of species	% of species
	A	B	C	D	E	F	G		
*Acinetobacter baumannii*	1	3	–	2	–	1	–	7	4.7
*Bacillus spp.*	3	7	–	3	5	10	7	35	23.3
*Burkholderia cepacia*	–	–	–	–	–	–	1	1	0.7
*Citrobacter freundii*	–	–	–	1	1	–	–	2	1.3
*Chryseomonas luteola*	1	1	–	–	–	–	–	2	1.3
*Escherichia coli*	–	1	6	3	1	–	–	11	7.3
*Klebsiella pneumoniae*	–	2	–	–	–	–	–	2	1.3
*Klebsiella ornithinolytica*	–	–	1	–	–	–	–	1	0.7
*Klebsiella oxytoca*	1	–	1	–	–	–	–	2	1.3
*Kluyvera* spp.	–	1	–	–	–	–	–	1	0.7
*Pantoea* spp.2	1	–	2	–	–	–	–	3	2.0
*Pantoea* spp. 3	–	–	–	–	–	–	2	2	1.3
*Pantoea* spp.4	–	–	1	–	3	–	–	4	2.7
*Pasteurella pneumotropica*	1	–	–	–	–	–	–	1	0.7
*Pseudomonas oryzihabitans*	1	1	–	1	1	–	–	4	2.7
*Serratia ficaria*	4	1	2	1	–	–	–	8	5.3
*Serratia marcescens*	1	–	–	–	–	–	–	1	0.7
*Serratia rubidaea*	1	–	–	–	–	–	–	1	0.7
*Shigella* spp.	1	–	–	–	–	–	–	1	0.7
*Staphylococcus aureus*	7	10	8	6	2	5	3	41	23.3
*Staphylococcus* spp.	3	4	2	5	3	1	1	19	12.7
*Stenotrophomonas maltophilia*	1	–	–	–	–	–	–	1	0.7
Total	27	31	23	22	16	17	14	150	100.0

Care units: A: Surgery; B: Maternity; C: Paediatrics; D: Medicine; E: Emergency; F: Operating Theatre; G: recovery room

*Staphylococcus aureus* (23.3%), *and Staphylococcus* spp. (12.7%) were present in all care units. *Pasteurella pneumotropica were one other GNB discovered*

## Discussion

Antibiotic resistance is a health problem with the expansion of multidrug-resistant strains such as Klebsiella pneumoniae [12–14]. Surface swabs of various hospital care units were used for the identification of different bacterial species in this study. From a total of 160 hospital surface samples, there were 65% positive bacterial cultures. The share of negative bacterial cultures is the result of environmental contamination control through cleaning and disinfection. The air quality of the room is also important, because particles suspended in the air inevitably end up landing on the surfaces. There was a statistically significant association between the Results of culture samples and different care units (*χ*2 = 12.732; *p* = 0.048).

We identified a high percentage of positive cultures in paediatrics (87.5%), followed by medicine (75%) (Table 1). Presence of health teams and visitors in the care unit, and their consequent contact with different patients, objects and surfaces imply the possibility of pathogen dissemination if the necessary precautions (especially hand washing) are not observed. However, other means may be involved in the transference of pathogens [15, 16]. The distribution of the results obtained from the culture of the samples in this study, reflected the level of efficiency of the treatment of the air of certain care units (operating theatre) and the quality of the cleaning or the disinfection of the hospital surfaces in general. These data encourage a strengthening of the principles of cleaning and disinfection of hospital surfaces.

There were microbes on all surfaces analysed, with *Staphylococcus aureus* and *Staphylococcus* spp. present in all positive samples across all care units in our study (Table 3). The constant presence of these two species in all departments could also indicate a lack of hygiene in this hospital environment. We found *S. aureus*, which is a commensal microorganism and opportunistic pathogen [17], in 27.3% of samples (Fig. 1). The presence of S. aureus is not surprising because of its’ opportunistic and ubiquitous nature [18, 19]. *Staphylococci* survives for days on some surfaces [20].

Methicillin-resistant *S. aureus* (MRSA) can survive for days in the hospital environment; 12 days on a table, and 9 days on tissue [21]. In a study reported in 2015 by Ouendo et al. in Benin, 20% of the bacteria responsible for healthcare-associated infections were *S. aureus* [7]. The significant presence of *S. aureus* in the care environment of the University Hospital of Abomey-Calavi / So-Ava in South Benin suggests the existence of infectious risk with the possibility of the appearance of methicillin-resistant strains.

Gram-negative bacilli (GNB) accounted for 36.7% (*n* = 55) in this study. Of the 150 species, Enterobacteriaceae accounted for 26.0%, followed by 15.0% NFGNB and 0.7% other GNB. Infections caused by GNB are specific because of the efficacy of these bacteria in the acquisition of genes that encode antibiotic resistance mechanisms [22]. In the hospital environment, these pathogens play an important role in the public health impact of healthcare-associated infections [23]. The detection rate of Enterobacteriaceae from all strains isolated in this study was 26.0%. This rate is comparable to the 23.5% obtained in 2007 in a hospital of Benin [24], and to the 25.03% reported in 2015 by a study in Tébessa (Algeria) in 2015 [25]. However, it was lower than the 67.33% obtained in 2008 on hospital environmental surfaces in Algeria [26]. These differences could be explained by the sample sizes.

In all 13 GNB genera isolated, there were Escherichia, Serratia and Pantoea. These genera can persist on dry surfaces for days [27]. In Enterobacteriaceae family, *Escherichia coli* 7.3% (*n* = 11) were the most frequent (Table 2). *E. coli* is a pathogen of healthcare-associated infections that poses problems in hospitals [28]. These include urinary tract infections, septicaemia, pneumonia, neonatal meningitis, peritonitis and gastroenteritis [17]. Escherichia coli ST131 est. un agent émergent résistant responsable d’infections des voies urinaires acquises dans la communauté et il est. important de contrôler sa propagation dans la communauté [29].

NFGNB are pathogenic bacteria with variable transmission capacities. Their identification at the species level is important for the management of patients suffering from healthcare-associated infections [30]. NFGNB are generally opportunistic pathogens that have been selected by repeated and prolonged antibiotic treatments [30]. In our study, *Acinetobacter baumannii* was the most frequently isolated NFGNB species at 46.7% (Fig. 2).

*A. baumannii* is an NFGNB present in the environment and commensal mucous membranes of man. This pathogen causes real therapeutic challenges because of its capacity to develop resistance to antibiotics [31–33]. The epidemic spread of *A. baumannii* is attributed to transmission on hands and to the prolonged survival of the microbe in the hospital environment [34]. This pathogen can persist from 3 days to 5 months [27], and survive under certain conditions for 4–5 months or more on dry surfaces [5]. The resistance of *A. baumannii* generates healthcare-associated infections with mortality up to 35% [35, 36].

## Conclusion

Healthcare-associated infections are a significant public health problem. The pathogens responsible for these infections are often present on hospital surfaces and equipment. It is important to identify these bacterial species in order to effectively prevent Healthcare-associated infections. Here, we report the pathogens present in the University Hospital of Abomey-Calavi /So-Ava in South Benin (West Africa). Some pathogens found evoke infectious risk and highlight the insufficiency of cleaning and the disinfection. Consequently, our results suggest an improvement in hospital hygiene and increased environmental disinfection is necessary.

## Abbreviations

API 20 E: Apparatus and methods of identification for the 20 characters of enterobacteria
BHB: Brain heart broth
EMB: Eosin methylene-blue
GNB: Gram-negative bacilli
GPB: Gram-positive bacilli
GPC: Gram-positive cocci
HAI: Healthcare-associated infections
ISO: International organization for standardization
MH: Mueller hinton agar
MRSA: Methicillin-resistant s. aureus
NFGNB: Non-fermenting gram-negative bacilli
SPSS: Statistical package for social sciences
UHC: University hospital center
